# Single-Wall Carbon Nanotube-Coated Cotton Yarn for Electrocardiography Transmission

**DOI:** 10.3390/mi9030132

**Published:** 2018-03-19

**Authors:** Yuliang Zhao, Yuying Cao, Junshan Liu, Zhikun Zhan, Xiaoli Li, Wen Jung Li

**Affiliations:** 1 School of Control Engineering, Northeastern University at Qinhuangdao, Qinhuangdao 066004, China; zhaoyuliang@neuq.edu.cn; 2 Department of Mechanical and Biomedical Engineering, City University of Hong Kong, Kowloon, Hong Kong 999077, China; 3 Key Lab of Industrial Computer Control Engineering of Hebei Province, School of Electrical Engineering, Yanshan University, Qinhuangdao 066004, China; caoyuyin@deakin.edu.au (Y.C.); liujunshan92@foxmail.com (J.L.); 4 Institute for Frontier Materials, Deakin University, Waurn Ponds, VIC 3216, Australia; 5 National Key Laboratory of Cognitive Neuroscience and Learning, Beijing Normal University, Beijing 100875, China; xiaoli_li@foxmail.com

**Keywords:** single-wall carbon nanotubes (SWNTs), conductive cotton yarns, flexible wires, wearable electronics, electrocardiography transmission

## Abstract

We fabricated a type of conductive fabric, specifically single-wall carbon nanotube-coated cotton yarns (SWNT-CYs), for electrocardiography (ECG) signal transmission utilizing a “dipping and drying” method. The conductive cotton yarns were prepared by dipping cotton yarns in SWNTs (single-wall carbon nanotubes) solutions and then drying them at room temperature—a simple process that shows consistency in successfully coating cotton yarns with conductive carbon nanotubes (CNTs). The influence of fabrication conditions on the conductivity properties of SWNT-CYs was investigated. The results demonstrate that our conductive yarns can transmit weak bio-electrical (i.e., ECG) signals without significant attenuation and distortion. Our conductive cotton yarns, which combine the flexibility of conventional fabrics and the good conductivity of SWNTs, are promising materials for wearable electronics and sensor applications in the future.

## 1. Introduction

Electrocardiography (ECG) is a widely accepted method to convey information of the heart’s electrical conduction system. It has been used to characterize heart condition and diagnose cardiovascular disease. Moreover, ECG signals can also be used to correlate the presence of any damage to the heart’s muscle cells and the effects on the heart from cardiac drugs. However, existing ECG signal collection methodologies are cumbersome and require systems that are bulky and not wearable. Hence, the development of wearable electronics, including portable and wearable ECG monitoring devices, in the last ten years has attracted the attention of researchers in the fields of personal health management and sports monitoring. In particular, polymer-based ECG-monitoring devices exhibit many desirable properties, which include being lightweight, flexible, durable, conformable, and portable [1,2,3,4]. 

In general, intrinsically insulating textiles are decorated with conductive materials to render them conductive. For example, conductive polymers, including polyacetylene, polypyrrole, and polyaniline, are used to modify cotton fabrics via a chemical polymerization process. The resultant conductive cotton fabric can be used in heating pads, flexible electrodes, or for static protection or sensors [5,6,7]. Moreover, researchers have utilized poly(3,4-ethylene dioxythiophene): poly(styrenesulfonate) (PEDOT: PSS) for ECG monitoring [2,8], and some breakthroughs in the study of brain activity [9] and long-term ECG monitoring [10] have been achieved using this material. However, the application of these conductive fabrics and polymers are often restricted by their complicated fabrication processes, such as micro-fabrication or chemical processing. For example, the fabrication of graphene-based textile electrodes starts with the preparation of graphene oxide, which requires multiple treatments with concentrated sulfuric acid (H_2_SO_4_), potassium permanganate (KMnO_4_), hydrogen peroxide (H_2_O_2_), and hydrochloric acid (HCl). Additionally, the resistance of a single or double PEDOT-PSS silk thread is typically in the order of MΩ·cm without the aid of chemical additives [11], which restricts the application of these threads for weak signal transmission. Therefore, a simple fabrication process for high-conductivity fibers that do not involve any dangerous chemicals or complicated processes is greatly in demand. 

Micro- or nano-metal particles represent another class of materials used for conductive coating [12]. For example, gold and silver nanoparticles, assembled as polymer brushes, have been used to enhance the conductivity of cotton fabrics. However, these materials are prohibitively costly and sometimes lack biocompatibility to be used in wearable devices.

Compared to conductive polymers and metal particles, carbon nanomaterials, such as carbon nanotubes (CNTs) or graphene, have outstanding material properties, such as excellent electrical conductivity, and other mechanical, electrochemical, electrical, and physical properties [3,13,14]. Carbon nanomaterials already have been used as conductive coating materials to fabricate conductive fabrics via a simple “dipping and drying” process [3,15,16]. Due to the hierarchical and porous structure of the fibers and microfibrils of cotton yarn, microfibrils easily “absorb” carbon nanomaterials [17] without requiring any surfactants or other surface treatments [18]. Compared to the commercial metal-based lead wires, these cotton-based lead wires are more comfortable and flexible, and could eventually have much lower cost. Ultimately, these cotton-based conductive yarns could be embedded or merged into clothes. In addition, high-conductivity yarns coated with CNTs will offer new applications in wearable electronics and sensors. Currently, however, the conductance of the cotton-based lead wires is not comparable to that of the metal-based lead wires. In this paper, we will discuss our work in demonstrating that single wall CNT-coated cotton yarns can be used to transmit weak human bioelectrical signals, such as ECG signals.

We note here that the toxicity of carbon nanotube has been debated for a long time, especially in the medical care field, where the toxicity of CNT have been studied in vivo [19,20,21]. Our ultimate purpose is to build wearable conductive fabric-based lead wires, which does not need to contact the human skin directly. However, the issue of how CNTs may affect the skin when in contact with human subjects should definitely be addressed, and many researchers have already embarked on research activities to address this question. For example, experiments on human volunteers and albino rabbits showed no association with skin irritation or allergens risks [22,23], but new studies still believe that deeper understanding is required for future medical acceptance of CNTs [24]. It is worth noting that, because the toxicity of CNTs often relate to their length, diameter, purity, production method, and functionalization, modifications of these factors may allow CNTs to be safe for human use [21]. For the envisioned applications of the single-wall carbon nanotube-coated cotton yarns (SWNT-CYs) discussed in this paper, we could develop an effective method to prevent the direct contact of CNTs to human skins by coating the SWNT-CYs with bio-compatible materials without affecting the overall conductivity of these fabrics. In this paper, we present a simple and controllable method of fabricating conductive cotton yarns via a “dipping and drying” process. method does not require complicated treatments compared to micro-fabrication and other dangerous chemical based methods. The electrical properties of SWNT-coated cotton yarns (SWNT-CYs), fabricated under different conditions, were investigated. These single SWNT-CY and multi-strand twisted SWNT-CYs were used to transmit the electrocardiography (ECG) signals of a young male (20 years old) subject. The collected ECG signals using our conductive fabrics are comparable to the signals collected using commercial metal-based lead wires for electrical signal detection. Details of the fabrication process for the SWNT-CYs and experimental results to acquire ECG signals from the human subject are presented in this article. 

## 2. Materials and Methods

### 2.1. Ethical Statement

The participation of the subject and use of the CNT products in the current study was approved by the Regional Ethical Review Board at Northeastern University at Qinhuangdao. Written informed consent was obtained from the subject before he participated in the study.

### 2.2. Materials Fabrication Process for Conductive Cotton Yarns

**Figure 1:**
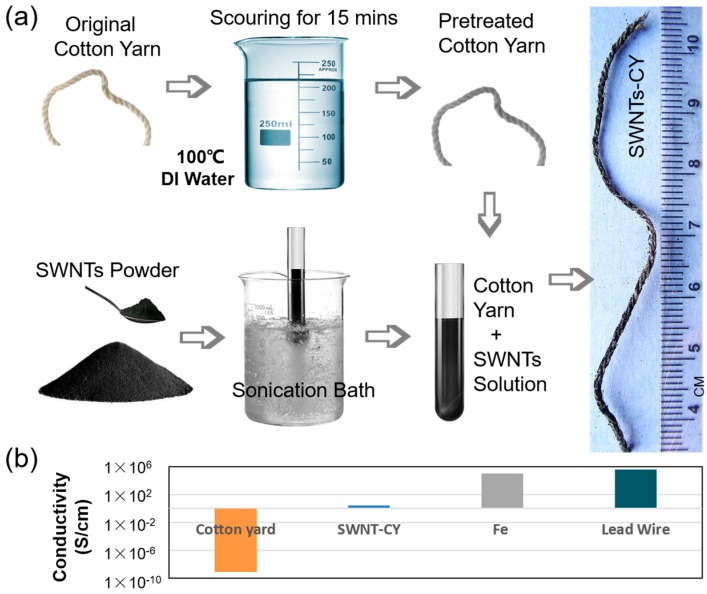
(**a**) Schematic illustration of the fabrication process for the conductive single-wall carbon nanotube (SWNT)-coated cotton yarns; and (**b**) the conductivity comparison of cotton yarn, carbon nanotube-coated cotton yarn (SWNT-CY), Fe [26], and commonly used lead wire.

In our study, we adopted the “dipping and drying” method, which is schematically illustrated in Figure 1, to fabricate conductive cotton yarns. To obtain a uniform coating, it was necessary to remove the original wax and other impurities on the surface of commercial cotton yarns [25]. Therefore, before being coated with SWNTs, the cotton yarns were immersed in hot water (100 °C) for 30 min, ultrasonically bathed for 15 min, and then left to air dry at room temperature. 

In the experiments described in this paper, we used SWNT powder purchased from Beijing DK Nano S&T Ltd., Beijing, China (Type: CNT100), which was >95% pure, and contained SWNTs with diameters between 1 nm and 2 nm and lengths between 5 μm and 30 μm (ASH < 0.5 wt %; specific surface area (SSA) > 450 m^2^/g; Electrical Conductivity > 100 S/cm). In order to prepare a well-dispersed colloidal solutions of SWNTs, 4–40 mg SWNT powder was dissolved in 2 mL deionized water in centrifuge tubes. Additionally, these samples were subjected to bath sonication for 30 min. The solutions were visually checked for larger agglomerates, without further microscopic verification. For the purpose of keeping the CNTs free from any surfactant residues, we sonicated the CNTs in pure deionized water. Since, the solutions were only stable for a short time, they were used immediately once prepared.

The purified cotton yarns were then dipped into the well-dispersed solution of SWNTs. The cotton yarns were observed to quickly absorb a large amount of SWNTs. After a period of contact, SWNTs and cellulose fibers could bond tightly together via van der Waals forces and hydrogen bonding. SWNT-coated fibers were then left to air dry for ~24 h to evaporate the water. Due to CNT uptake, the weight of the cotton yarns increased by 1–3 mg (8–20% of the whole weight of SWNT-CYs), while the diameter does not have significant change. Thus, SWNT-coated cotton yarns (SWNT-CYs) could be successfully prepared via “one dip”.

### 2.3. Measurement of Electromechanical Properties

To evaluate the electrical conductivity of SWNT-CYs, the SWNT-CYs were loaded into a clip, which was fixed to an electrically insulated board, to straighten the cotton yarns. All specimens were tested at a length of 6 cm. A tensile tester (Instron 5848, Norwood, MA, USA) was used to apply a tension and stretch the specimen, and a source-meter (Keithley 2400 SourceMeter, Norwood, MA, USA) was used to measure the conductivity of each SWNT-CY via the cyclic voltammetry (CV) method. 

### 2.4. Electrocardiography (ECG) Monitoring

**Figure 2:**
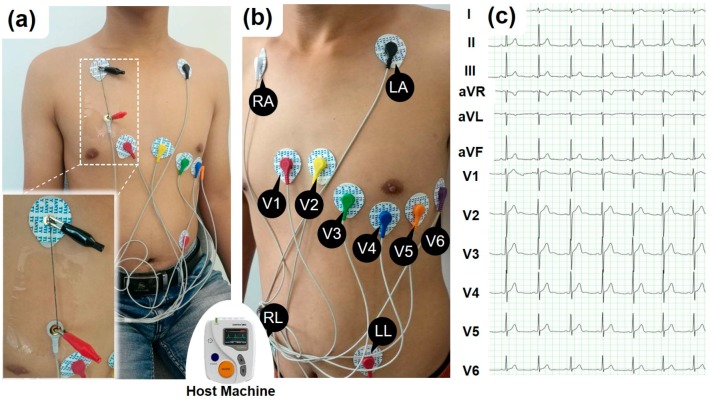
The experimental methods and setup of recording and transmitting human ECG signals using the SWNT-CYs. (**a**) The subject (20 years old, male) is sitting up straight on a chair during the test. Two alligator clips are used to fix the ends of a “wire” made of SWNT-CYs to electrode and the original wire (shown in the inset figure). The CNT-CYs “wire” does not contact the subject’s skin directly during the ECG data collection process; (**b**) the 12-Lead ECG electrodes placement on a volunteer based on the specification of the Dynamic ECG Systems; and (**c**) the ECG signals of these 12 channels measured via the original commercial lead wires.

Figure 2 illustrates the methodology of ECG measurement based on a commercial product (TLC6000, Contec Medical Systems Co., Ltd., Hebei, China), which is used in medical institutions. According to its specifications, a 12-lead was adopted to record the ECG signal. Disposable AgCl electrodes (CH50B, Cathay Manufacturing Corp, Shanghai, China) were placed on the skin of the subject. Figure 2a,b show the locations where the electrodes were placed. The 12-lead ECG signals of a 20 years old male subject measured through the lead wire are shown in Figure 2c. During the experiment of ECG signal transmission, the conductive cotton yarn is added between the electrode and the host machine, which is shown in the Figure 2a. For instance, in the measurement of V1 channel, the electrode (V1) was connected to one end of an SWNT-CY sample, while the other end was connected to the host machine via the original lead wire, which cannot be detached from the host machine. This machine can continuously record ECG waveforms for 48 h, analyze the ECG waveform by a proprietary software, and filter most of the noise of the ECG signals. The ECG signals, shown in Figure 2c, was produced and filtered by the host machine and then shown by the proprietary interface software without any modification. 

## 3. Results

### 3.1. Morphology of Cotton Yarn before and after SWNT Coating

**Figure 3:**
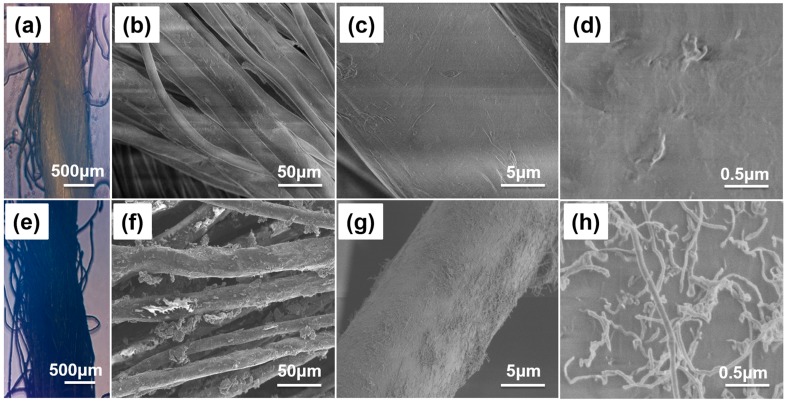
Microscope and SEM images of the hierarchical structure and surface morphology of cotton yarns before and after SWNT coating. (**a**,**e**) are optical images of two-strand twisted pristine cotton yarn and SWNT-coating cotton yarn; (**b**–**d**) SEM images of the hierarchical structure and morphology of pristine cotton yarn (no coating); (**f**–**h**) SEM images of the hierarchical structure and morphology of SWNT-coated cotton microfibrils.

A two-strand twisted cotton yarn made of cellulose fibers was utilized as the matrix structure for SWNT coating. The contrast in the hierarchical structure and surface morphology of pristine cotton yarn (Figure 3a–d) and SWNT-CY (Figure 3e–h) are shown on different length scales. 

In Figure 3g,h, a multidirectional coating of SWNTs on the surface of microfibrils was observed. Numerous conducting pathways were formed on the surface of cotton microfibrils that resulted in conductive cotton yarns. The uniformity of the SWNT solution is crucial in determining the uniformity and polarity of SWNTs coated onto the surface of cotton microfibrils, which ultimately affects the conductivity of SWNT-CYs. 

### 3.2. Electrical Conductivity Properties of SWNT-CYs

#### 3.2.1. Conductivity Control of SWNT-CYs

**Figure 4:**
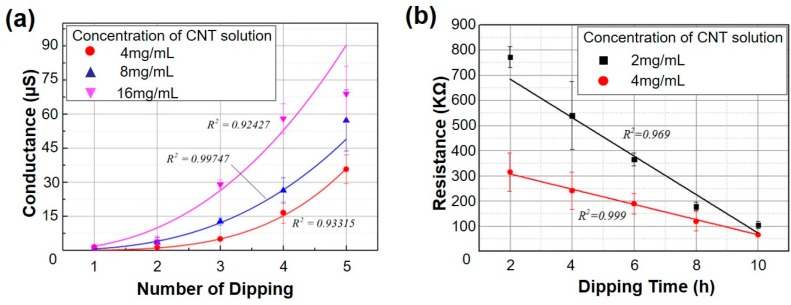
(**a**) The relationship between the conductance of SWNT-CY and the total number of dips of the cotton yarn into the SWNT solution at concentrations of 4 mg/mL, 8 mg/mL, and 16 mg/mL (error bars show the standard deviations of five examples); (**b**) The relationship between the resistance of SWNT-CY and dipping time in the SWNT solution at concentrations of 2 mg/mL and 4 mg/mL (error bars show the standard deviations of five examples).

Three parameters in the fabrication process of SWNT-CYs determine the conductivity of the SWNT-CYs: the concentration of the SWNT solution, the duration of incubation during each dip, and the total number of dips. Figure 4 show effects of the concentration of the SWNT solution, the duration of incubation during each dip, and the total number of dips on the conductivity of two-strand twist SWNT-CYs. As shown, the conductivity of SWNT-CYs increased exponentially with the total number of dips. 

The dipping time in Figure 4b denotes the duration that the cotton yarn was immersed in the SWNT solution. We tested the effects of dipping time on the conductivity of SWNT-CYs with SWNT solutions at 2 mg/mL and 4 mg/mL in a single dipping process. We investigated dipping times from 2 to 10 h. Figure 4b shows that conductivity increased with the dipping time.

In general, the conductivity of SWNT-CYs can be enhanced by increasing the concentration of the SWNT solution, dipping time, and total number of dips, which leads to the absorption of more CNTs onto the cotton yarn fibers. Thus, the conductivity of SWNT-CYs can be effectively controlled by changing these parameters. 

#### 3.2.2. Conductivity and Mechanical Characteristics

**Figure 5:**
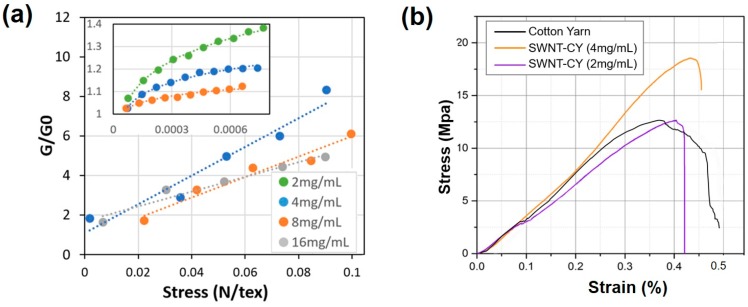
(**a**) The relationship between the conductance of SWNT-CYs (relative change, G/G0) and the tension applied to the SWNT-CYs dipped in SWNT solutions of different concentrations. The “tex” is a unit of textile measurement, which is defined as the mass in grams per 1000 m, i.e., describes the linear mass density of fibers. The inset data-plot shows the relationship between these two values when the stress is below 0.001 N/tex; (**b**) Stress-strain curves of cotton yarn (black line) and SWNT-CYs (yellow line, 4 mg/mL; purple line, 2 mg/mL) in the fracture tests.

Figure 5a shows the relative conductance change of SWNT-CYs in the stretched (G) and relaxed (G0) states. These tested SWNT-CYs are single yarns coated with SWNT solutions at 2 mg/mL, 4 mg/mL, 8 mg/mL, and 16 mg/mL. The stress-strain relationship of twisted SWNT-CY was studied on a tensile tester (5848, Instron, Norwood, MA, USA) and shown in Figure 5b. The loading forces starting from 0 and were applied increasingly until the SWNT-CYs broke. The results show that the yield strength of cotton yarn increased after SWNT coating. Meanwhile, the SWNT-CYs still maintain their flexibility and softness.

The relative rate of change of conductivity with respect to tension is greater for the single SWNT-CYs modified with a lower concentration of the SWNT solution (4 mg/mL) than for the single SWNT-CY modified with a higher concentration of the SWNT solution (8 mg/mL). Additionally, the relative rate of change of conductivity with respect to tension is less for multi-strand twisted SWNT-CYs than for single SWNT-CYs. The strain, i.e., loading on the twisting structure of SWNT-CYs, compresses the SWNT-CYs laterally. Hence, straining the SWNT-CYs may lead to better contact between the CNTs, which would consequently promote the formation of more efficient conducting pathways along the yarns. Meanwhile, the same amount of pulling force leads to less tensile strain in a multi-strand yarn than in single yarns. Hence, the lateral compression in a multi-strand yarn due to the twist is less. This may explain the resulting lower relative rate of change of conductivity for multi-strand twisted SWNT-CYs than for single strand SWNT-CYs.

### 3.3. Electrocardiography (ECG) Transmission

The electrocardiographic signals, which is weak and sensitive to various disturbances, is measured in medical clinics using highly specialized and sensitive equipment. At the body surface, the amplitude of cardiac signals typically varies from 400 μV to 2.5 mV [27,28]. The ECG is composed of a series of wave groups, which reflect the different stages of the ECG signals and include the P-wave, QRS complex, T-wave, and U-wave. Figure 2c shows a group waveform of a 12-lead ECG signals. The signal of each lead has a different typical waveform. The duration of each wave and interphase intervals are meaningful for diagnostic purposes. 

**Figure 6:**
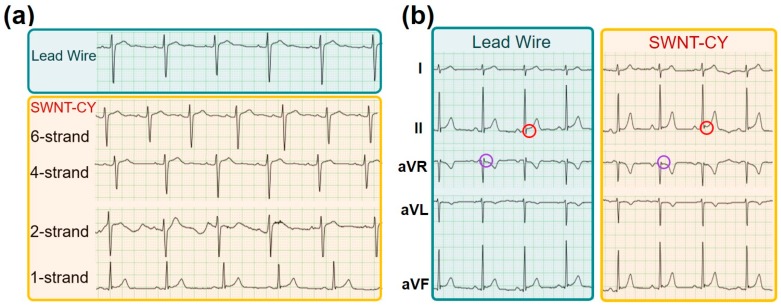
(**a**) ECG signals of V1-lead recorded from the same subject (20 years old, male) using commercial lead wires (area in light blue) and SWNT-CYs (area in light yellow, connecting the V1 electrode and the host machine); and (**b**) a comparison of ECG signal recorded by metal-based lead wires and 4-strand SWNT-CYs (connecting the RA electrode and the host machine). All these ECG signals were obtained from the empirical ECG signals acquired by the pre-installed filter of the measurement system. The gap between two horizontal green lines represents 500 mv, while the distance between the vertical green lines represents 200 ms.

To investigate the ability of the conductive yarns to transmit weak bio-electrical signals at the body surface, we recorded the ECG signal of a 20 years old young male subject. The experimental configuration of collecting the ECG signal is shown in Figure 2. To validate the performance of the setup, we initially recorded the ECG signal using a commercial metal-based lead wire. The result, as shown in Figure 2c, indicates that the weak signal of the ECG could be successfully captured and transmitted using our experimental setup. These typical wave groups could be easily identified in Figure 2c and Figure 6 (light blue area). Although the amplitude of the noise varies, after filtering by the host machine, the ECG signals were clearly observed. 

After the standard measurements using the ECG measuring system, we then recorded the ECG signal using the SWNT-CYs in place of the commercial lead wire without any other changes to the recording setup. The conductance of a single-strand SWNT-CY we used is greater than 4 μS/cm. Figure 6a shows the results of ECG signals of V1-lead recorded using a single-strand, two-strand, four-strand, and six-strand twisted SWNT-CYs, respectively, (light yellow area) compared to that recorded using the original metal-based lead wire (light blue area). In the comparison, it is obvious that the waveform of the ECG signal measured by a single-strand SWNT-CY is incorrect. The ECG signal measured by the two-strand SWNT-CYs show some unwanted turbulence and noise. Meanwhile, the ECG waveforms measured by four-strand and six-strand SWNT-CYs are similar to the standard waveforms measured by the commercial lead wires. 

As stated earlier, we connected the SWNT-CYs “wire” to the RA electrode, which was then connected to the host machine (as shown in Figure 2a) during the experiments. Since this configuration of electrical connection has little impact on ECG signals of V1 to V6 leads, we only show the comparison of the ECG signals of I, II, aVR, aVL, and aVF leads as transmitted by a lead wire and a SWNT-CYs in Figure 6b. For this example data shown in Figure 6b, a four-strand SWNT-CYs “wire” was used. As shown, the ECG signals from both the SWNT-CYs and the metal-based lead wire had similar waveform profiles. The peak values of the QRS complex were nearly identical. Moreover, the details of these waveforms (marked by red and purple circles) have a little distortion. In summary, after the filtering by the host machine, the noise level of the ECG signal recorded with the multi-strand twisted SWNT-CYs was similar to that of the ECG signal recorded with the metal-based lead wire. Compared to the single-strand SWNT-CYs, multi-strand twisted SWNT-CYs transmitted ECG signals more similar to that of the commercial lead wires. Both Figure 6a,b demonstrate that SWNT-CYs can be applied for transmitting ECG or possibly other weak human physiological signals. 

## 4. Conclusions

We have developed a simple, effective, and controllable process for fabricating conductive cotton yarns, which can be used as flexible conducting “wires” to transmit weak bioelectrical signals. The multi-directional coating of SWNTs on the microfibril surface assembles themselves into conducting pathways. Various experiments were performed to investigate the electrical conductivity and transmission performance of single SWNT-CY and multi-strand twisted SWNT-CYs. We also demonstrated that these SWNT-CYs could be used as traditional conductors for transmitting weak ECG signals of the human body in place of commercial metal-based lead wire for electrical signal detection. We have demonstrated that the conductivity and transmitted signal strength could be comparable to that of metal-based lead wires. By combining favorable characteristics of comfortability, good conductivity, low-cost, and ease of fabrication, we think that the SWNT-CYs is a promising material for broad applications in wearable sensors and electronics.
